# The Impact of Neutrons in Clinical Proton Therapy

**DOI:** 10.3389/fonc.2015.00235

**Published:** 2015-10-21

**Authors:** Uwe Schneider, Roger Hälg

**Affiliations:** ^1^Institute of Physics, Science Faculty, University of Zürich, Zürich, Switzerland; ^2^Radiotherapy Hirslanden, Zürich, Switzerland

**Keywords:** proton therapy, neutrons, second cancer

## Abstract

In proton therapy, high-energy proton beams cause the production of secondary neutrons. This leads to an unwanted dose contribution, which can be considerable for tissues outside of the target volume regarding the long-term health of cancer patients. Due to the high biological effectiveness of neutrons with regard to cancer induction, small neutron doses can be important. Published comparisons of neutron dose measurements and the corresponding estimates of cancer risk between different treatment modalities differ over orders of magnitude. In this report, the controversy about the impact of the neutron dose in proton therapy is critically discussed and viewed in the light of new epidemiological studies. In summary, the impact of neutron dose on cancer risk can be determined correctly only if the dose distributions are carefully measured or computed. It is important to include not only the neutron component into comparisons but also the complete deposition of energy as precisely as possible. Cancer risk comparisons between different radiation qualities, treatment machines, and techniques have to be performed under similar conditions. It seems that in the past, the uncertainty in the models which lead from dose to risk were overestimated when compared with erroneous dose comparisons. Current risk models used with carefully obtained dose distributions predict a second cancer risk reduction for active protons vs. photons and a more or less constant risk of passive protons vs. photons. Those findings are in general agreement with newly obtained epidemiologically results.

## Introduction

During proton therapy, neutrons are produced. This is known since protons are used for applications in radiation therapy. It is also known that the neutron absorbed dose is small. However, neutrons are highly biological effective and thus even a small absorbed dose might cause side effects in the patient, the most severe of which is the induction of a second primary cancer. For this reason, since the 1990s, the following main approaches to quantify the neutron absorbed and equivalent dose in radiotherapy patients include:

(i) Neutron, proton, and photonuclear cross-sections and neutron kerma coefficients for radiation therapy were determined based on experimental data and nuclear model calculations. Such data permit calculations of absorbed dose in the body from therapy beams, and through use of kerma coefficients allow absorbed dose to be estimated for a given neutron energy distribution. Most work in the beginning was done by Chadwick (1) and was extended afterward by many other authors.
(ii) Monte Carlo simulations of the neutron dose and neutron energy spectra were performed for typical proton therapy facilities. First work was published by Agosteo et al. (2) and Siebers (3) and further work was published since then, including very detailed simulations of proton therapy beam lines including realistic patient geometries.
(iii) Measurements and calculations of the quality factor of neutrons with the endpoint of cancer induction (and/or chromosomal aberrations, clonogenic survival, neoplastic transformations, etc.) were performed. Many studies were conducted starting in the 1970s. Much work was motivated by space radiation research and the A-bomb survivor analysis [e.g., Ref. (4–6)]. Later, also nanodosimetric measurements were used to characterize the quality factor of neutrons produced by proton beams (7).
(iv) Measurements of neutron absorbed dose and neutron dose equivalent at proton therapy beam lines were executed. First, measurements on passive beam lines were undertaken by Binns and Hough (8) and Yan et al. (9) and for active beam lines by Schneider et al. (10). Since then, a large number of neutron dose measurements were reported including several beam lines, in patient measurements, as well as measurements in treatment rooms, including a variety of dosimeters and set-ups including neutron energy spectra measurements. In addition, analytical methods were developed to determine neutron dose equivalent (11).

The resulting measured or simulated neutron dose distributions were used to estimate the risk for radiotherapy patients to develop secondary malignancies. Two strategies were usually applied. Either the neutron dose distribution was viewed as an additional dose burden to the patient, independently of the delivered dose to treat the tumor. As the neutron doses are usually low, radiation protection models were used to convert dose to risk. Another possibility is to combine the neutron dose with the dose distribution delivered by the therapy protons. The resulting dose levels are then much larger than the scope of radiation protection models and thus newly developed RT-risk-models were used to study the impact of the additional neutron dose. The latter models include, therefore, also the impact of integral dose changes on cancer risk. In the year 2006, two reports (12, 13), using these concepts, were published. The two strategies which were used to estimate second cancer risk came to completely contrary conclusions. Hall (12) estimated the risk of second malignancies by analyzing the stray and neutron doses alone and concluded that passive proton therapy would result in up to 20 times more second cancers than conventional photon radiotherapy. On the other hand, Schneider et al. (13, 14) determined cancer risk by analyzing the complete dose distribution including the energy deposited by primary protons and neutrons. They found for active proton therapy a decrease in second cancer risk. For passive proton therapy, by scaling the neutron dose, the risk was more or less the same when compared to conventional photons. This resulted in a heavily discussed controversy about the future of proton therapy.

In this report, we highlight the controversy about the impact of the neutron dose in proton therapy, which is critically discussed and viewed in the light of new epidemiological studies. The aim of this work is not to provide a review summarizing the current knowledge of neutron dose measurements, calculations or simulations, and the resulting cancer risk estimates.

## Measured and Simulated Neutron Dose

**Figure 1:**
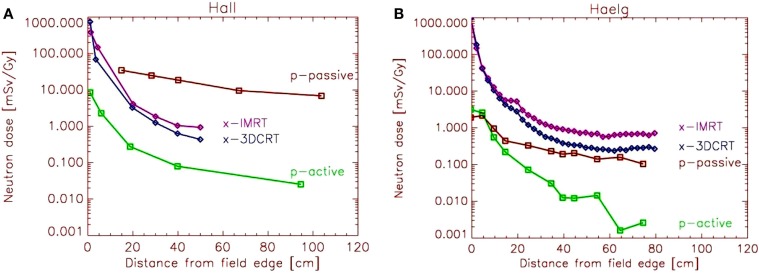
**(A)** Eric Hall’s comparison (12) of neutron dose equivalent per treatment Gray as a function from the distance of the field edge. Measurements of several researchers were combined by scaling the neutron dose. **(B)** Comparison of neutron dose equivalent by Hälg et al. (13). The measurements were performed under the same conditions for each treatment modality.

It is of importance that dose and risk comparisons with regard to radiation quality and treatment technique are performed using the same phantom or patient, the same experimental equipment and is based on the same clinical indication. Treatment planning should be performed using the same dose constraints for target and normal tissues. If measurements of different experimental set-ups are compared very easily, apples and oranges are compared. Figure 1A shows a dose comparison from Ref. (12), where measurements obtained by different researchers were compared.

On the basis of Figure 1A, Hall (12) has drawn the conclusion that IMRT with photons would double the incidence of solid cancers in long-term survivors and passive proton therapy would result in up to 20 times more second malignancies. The dose scaling of the different experimental results, which led to Figure 1A, were highly questioned and resulted in the exchange of several letters to the editor.

A fair comparison of stray doses is shown in Figure 1B, which was obtained using for the investigated treatment modalities and techniques the same phantom and the same treatment indication (15). For the measurements at the passive proton therapy, beam line compensators were used which were produced specifically for this case. As a result, the distribution of the neutron dose of scattered protons is completely different when compared to the published data of Hall (12) reaching two orders of magnitude at 40 cm distance from the field edge. Clearly, the conclusion drawn by Hall was wrong, as he used erroneous stray dose estimates. Using the dose results of Figure 1B, one would expect for passive proton therapy approximately the same amount of neutron dose than photon stray dose produced by conventional 3D conformal radiotherapy. However, it should be noted here that the neutron measurements are related to large errors and that the quality factor for cancer induction is not well known. In addition, the effect of prompt gamma radiation in proton therapy was not considered.

## Models of Second Cancer Induction

### Estimates Based on Dose Comparison

Using simple dose comparisons for risk estimates by applying data, as shown in Figure 1, can be unsafe for two reasons. The decrease of dose as a function from field edge is exponential or sometimes even more than exponential. Since risk is both a function of dose and irradiated volume, it is very important to analyze carefully the shape of the dose curves close to the target volume. For example, when using IMRT techniques with photons, the dose far away from the field edge might by larger when compared to 3DCRT. However, the dose close to the field edge is lower for IMRT techniques. The reason for this is that IMRT produces less phantom scatter, which is the major stray dose component close to the target. Although the affected volume might be small, the dose at around 10 cm from the field edge can be more than a magnitude larger than far away from the treatment field.

The second reason is that we do not get an idea about the full 3D-dose distribution by analyzing only certain components of the dose, e.g., the neutron dose. Generally the dose distribution can be separated into two parts. The in-field dose is created by particles impinging on the patient through the opening of the beam aperture. This includes in-field scattering mainly produced by Compton scattering (photons) and multiple Coulomb scattering or inelastic nuclear interactions (protons and ions). The out-of-field dose is generated by phantom scatter and radiation scattered by the treatment head, leakage radiation through the collimators and neutrons, and prompt gammas produced either in the machine or the patient.

For a reliable risk estimate of the patient, it is required to study the deposited energy of all components. In doing so, the characteristics of dose deposition of the different radiation qualities are taken into account. If, for example, photons are compared to protons, the integral dose in the highly irradiated volumes is always a factor of 2–3 lower for protons, independently of the treatment technique (16). That must have an impact on cancer induction and cannot be neglected.

In summary, relative risk estimates using comparisons of dose distributions are possible. However, it is essential that the correct dose distributions are compared, including all relevant stray dose components. The comparisons must be obtained by selecting carefully the same conditions for all treatment types in questions if dose measurements or simulations are performed. Currently, measurements as well as analytical or Monte Carlo simulations can predict stray doses with a precision of around 20–50%.

### Estimates Based on Risk Models

Simple models to predict risk of radiation-induced cancer for radiotherapy dose levels are based on conventional concepts from radiation protection, i.e., ICRP (17) or BEIR (18). These models are based on the linear approximation of the risks of the Atomic-bomb survivors and use effective dose (the tissue-weighted sum of the equivalent doses in all specified tissues) for risk estimation. Basic risk factors are usually modified by a dose and dose-rate effectiveness factor (DDREF) for the application to low dose-rates. The linear model is only valid for doses up to around 1–2 Gy and as such, is in general, not applicable to complete radiotherapy dose distribution.

Radiation protection models can be safely applied exclusively to the dose originating from scatter radiation. In principle, the linear model is applied to very low doses with a threshold of around 100 mSv. The threshold represents the maximum applied scatter dose during a typical radiotherapy treatment (Figure 1B, if scaled to a typical RT dose). As for such estimates, only the out-of-field-dose is considered, but the in-field dose distribution completely neglected, cancer risk is not a function of the integral dose, but proportional to the amount of scatter dose. As a consequence, such studies result in an estimated increase of cancer risk of modern radiotherapy techniques (12). The reason for this is the larger amount of scatter, leakage, and neutron dose of those treatment modalities compared to conventional treatment techniques. While in such situations the application of radiation protection concepts may be appropriate, since exclusively the low doses are investigated, the main disadvantage of such an approach is that the in-field dose distribution (>100 mSv) is completely neglected. Thus, risk estimates based on scatter dose would only include second cancer induction far away from the treated side. It is reported, however, that only around 20% of all radiation-induced malignancies are found far away from the treated volume (19).

In summary, radiation protection models should be used only with extreme care for risk estimates in radiotherapy since they are developed exclusively for low dose. When applied to scatter radiation, such models can predict only a fraction of observed second malignancies.

**Figure 2:**
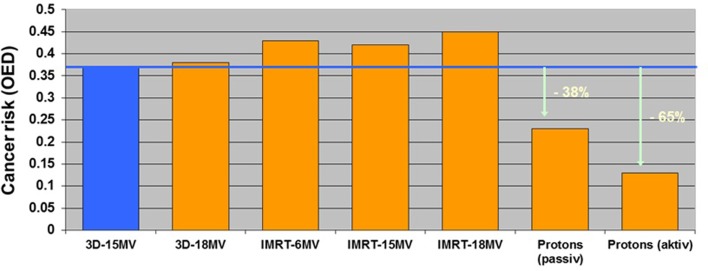
**Modeled second cancer risk after radiotherapy of the prostate relative to a historic radiation treatment (four-field-box in blue)**. The data were taken from Ref. (14) and updated with better dose measurements (15). Prostate cancer was chosen for comparison with the epidemiological study of Chung et al. (21) as their patient cohort consisted mainly of prostate patients.

It is also possible to take for cancer risk estimates the complete 3D-dose distribution (in- and out-of-field) into account by using semi-empirical models of cancer induction. Such models include the effect of dose fractionation and represent the dose–response relationships more accurately. The involved uncertainties are still huge for most of the organs and tissues. A major reason for this is that the underlying processes of the induction of carcinoma and sarcoma are not well known. Most uncertainties are related to the time patterns of cancer induction, the population specific dependencies and to the organ-specific cancer induction rates. For radiotherapy treatment plan optimization, these factors are irrelevant as a treatment plan comparison is performed for a patient of specific age, sex, etc. If a treatment plan is compared relative to another, a precision of around 10% can be achieved (20). Such a model was used in Ref. (13) for cancer risk estimates after prostate radiotherapy by using the complete 3D-dose distribution including stray dose estimates. It was found that the additional dose of neutrons during proton radiotherapy is balanced by the integral dose advantage of proton beams. The predicted risk of passively scattered protons is, thus, slightly lower than of photon 3DCRT. Actively applied proton beams resulted in more than 50% reduced risk prediction relative to 3DCRT (Figure 2).

## Qualitative Comparison of Model Predictions with Epidemiological Findings

In 2006, when contradicting model results were published (12, 13), no epidemiological study regarding second cancers after proton therapy or IMRT was available. The users of proton therapy machines, and also the clinicians who were using photon IMRT, were confused about which models to believe. On the one hand, Eric Hall (12) predicted, by using stray dose comparisons, a 2- and 20-time larger second cancer risk for IMRT and passive proton therapy, respectively. On the other hand, risk models that were applied to the complete dose distribution of a patient predicted more or less the same risk for IMRT using 6 MV photons and passive proton therapy (13, 14).

In 2013, the first epidemiological study on a comparison of second cancer risk between a photon and proton-treated group was published by Chung et al. (21). They found that the use of proton radiation therapy using passively scattered protons was not associated with a significantly increased risk of secondary malignancies compared with photon therapy. Although they state, that longer follow-up of these patients is needed to determine if there is a significant decrease in second malignancies, they found an adjusted hazard ratio of 0.52 [95% confidence interval, 0.32–0.85] of protons vs. photons. These first epidemiological results strongly suggest that the exaggerated risk estimates of Ref. (12) which were based on a faulty stray dose comparison were wrong.

In a study published 2014, Sethi et al. (22) examined in-field and out-of-field cancer incidence in proton vs. photon-treated patients with retinoblastoma. In-field cancer was significantly higher in photon-treated patients. With an ~7-year median follow-up, the incidence of out-of-field cancer did not significantly differ in the proton- vs. photon-treated patients. These results are in accordance with the integral dose advantage of protons vs. photons and the comparable stray doses for scattered protons and 3DCRT, as shown in Figure 1B.

## Conclusion

Most criticisms of cancer risk estimates are usually given to the uncertainties of risk models, which lead from dose to second cancer risk. We are concerned that there is too little thought being given to the very simple ideas on which cancer risk models are based upon and too little objections about accepting the implications of such models. However, even more important are the errors and uncertainties in the dose distributions, which are the basis of risk modeling. If the dose is wrongly quantified, like in Figure 1A, this leads inevitably to wrong risk estimates, regardless of the quality of the used risk models. It is also important to always take the full dose distribution into account and not only parts of it. This is of particular importance when photon therapy is compared to proton therapy, as the integral dose advantage of proton therapy in the highly irradiated volumes can be balanced by the neutron dose in the areas distant from the irradiation fields. Unfortunately, researchers are often using over-simplified dose estimates, by applying risk models e.g., to dose distributions obtained from radiotherapy treatment planning systems.

In summary, if carefully obtained dose distributions are used with appropriate risk models to predict second cancer for radiotherapy patients, a reduction for active and passive proton therapy is predicted when compared to photons. Those findings are in general agreement with newly obtained epidemiologically results. The estimates performed by Hall (12) resulting in an order of magnitude enhanced risk of passive proton therapy are contradicted by the findings of the epidemiological studies and various risk estimates for radiotherapy patients.

In the future, it is important to gain more knowledge on the RBE of neutrons with regard to cancer induction. It is necessary to study RBE for tumor induction as a function of neutron dose, energy, dose-rate, tissue type, and size of the exposed patient. Currently, the EU project ANDANTE (23) is exploring the question of neutron RBE.

More research is also necessary to improve the precision of out-of-field neutron dose calculations including the energy spectra. This could make whole-body dose calculations available for risk estimates of individual radiotherapy patients.

## Conflict of Interest Statement

The authors declare that the research was conducted in the absence of any commercial or financial relationships that could be construed as a potential conflict of interest.
